# Microscopical Examination of Tissues during Operation

**Published:** 1907-10-19

**Authors:** 


### MICROSCOPICAL EXAMINATION OF TISSUES DURING OPERATION.

It happens not infrequently in surgical cases that there is some doubt even at the time of operation as to whether a particular lesion is benign, moderately malignant, or very malignant. The extent of the operative measures taken must clearly be much influenced by the character of the particular tumour that is being dealt with. If there is even a possibility of its being malignant wide removal will be done if it is practicable. It is a general feeling that it is better to err in the direction of doing too much in the case of a benign lump than in that of doing too little when the trouble is malignant; and if there were no means at hand of deciding otherwise this rule would doubtless be a good one. It is not scientific and exact, however; and sometimes for fear of doing an extensive operation for a condition which requires much less to be done, two operations are performed instead of one. At the first only a portion of the mass is cut out, and the patient is returned to bed to await the result of histological examination of a section under the microscope; if the section shows malignant disease a second operation has to be undertaken for its removal as radically as possible.

The necessity for these double operations in somewhat doubtful cases no longer exists. Surgeons can now have a skilled microscopist to assist them at the time of the first operation; by the aid of a freezing microtome it is possible to prepare and examine a microscopical section in so short a time that the anæsthesia of the patient need not be interrupted; and the surgeon can continue the operation, guided as to what he shall do in the patient's best interests by the report upon a section made from a piece of the tissue just excised by him.

The method is briefly as follows:—A good freezing microtome, with all its accessories, together with the necessary dishes and reagents, are all got ready before the anæsthetic is begun. The microtome should be firmly fixed on a strong table, and the microscopist should be sure that everything is in its proper place. This ensures that no time will be wasted when once the process is begun. The patient having been anæsthetised the surgeon rapidly excises a portion of the tissue he wishes examined. The selected piece is quickly shaped with a knife or scissors, and placed directly on to the brass disc of the ether-freezing microtome, and these covered with the gum solution. The ether-bellows are worked by one hand, and the tissue and gum rapidly frozen to a hard uniform mass. The special razor in its carrier is quickly passed a few times across the frozen tissue, sections of the requisite thinness being thereby cut with ease. The sections are transferred *en masse* to a dish of cold water, or, better still, normal saline solution; they are separated from one another with the end of a glass rod, and the one which seems the best is quickly lifted out, either on the rod or on a microscope slide, and dipped into pure methylated spirit for a moment. From the spirit it is similarly transferred to another larger dish of cold water or normal saline, where it immediately flattens out by reason of the currents set up by the mingling of the spirit in and on the section with the water around it. A clean microscope slide is dipped obliquely under the flattened out section; the latter, held in position by a needle, is lifted out on the slide; the excess of water is allowed to drain off for a moment, and then a drop or two of the stain is allowed to fall directly on to the section.

Löffler's methylene blue is used as a rule, but the choice of stain naturally remains with the microscopist. The methylene blue is convenient on account of the rapidity with which it stains. In about five seconds—that is to say, almost as quickly as it can be done—a thin cover-glass is carefully lowered on to the staining section, and gently pressed down with blotting paper. The latter absorbs the surplus stain as quickly as it is expressed from under the cover-glass, so that without further preparation the section is ready for examination under the microscope. If a few minutes longer can be allowed the water in the section can be replaced with glycerine, and the stained tissue mounted in the latter. The cells are clearer in this case and easier to examine and report upon than when they are examined in plain water. The surgeon receives the verbal report of the microscopist, and continues the operation with certain knowledge of the condition he is dealing with.

